# Spirituality and Religiosity of Internal Medicine Physicians in the USA: Results from a National Survey

**DOI:** 10.1007/s11606-025-09651-y

**Published:** 2025-06-25

**Authors:** Kristin M. Collier, M. Todd Greene, David Ratz, Rachel Ehrlinger, Sanjay Saint

**Affiliations:** 1 Department of Internal Medicine, University of Michigan Medical School, Ann Arbor, MI USA; 2 Patient Safety Enhancement Program, University of Michigan and Veterans Affairs (VA) Ann Arbor Healthcare System, Ann Arbor, MI USA; 3 Veterans Affairs Ann Arbor Healthcare System, Ann Arbor, MI USA

**Keywords:** Spirituality, Religiosity, Burnout, Protective factors

## Abstract

**Background:**

Medicine as a profession is steeped in meaning. Spiritual and religious practices are one way in which providers make meaning in their lives and therefore in their work. Recent attention has focused on the religious and spiritual commitments of physicians as they relate to topics such as self-care, physician conscience, and how these beliefs impact clinical practice.

**Objective:**

To assess the religious and spiritual beliefs of internal medicine physicians and the degree to which these beliefs are associated with burnout.

**Design:**

A national, cross-sectional survey of internal medicine physicians.

**Participants:**

Between June 2023 and May 2024, surveys were sent to 1421 randomly selected internal medicine physicians practicing in the USA identified through the American Medical Association membership database.

**Main Measures:**

The survey included 42 questions designed to assess factors hypothesized to influence physician well-being and professional burnout. We also collected physician demographic data and assessed burnout via the Maslach Burnout Inventory.

**Key Results:**

A total of 629 (44.3%) completed a survey. Almost 70% of the general internists who responded endorsed belief in God or a higher power, and approximately half endorsed praying privately at least once a week at a place other than a place of worship and had a belief in life after death. Black respondents had four times greater odds of believing in God compared with non-Black respondents (OR = 4.14, 95% CI = 1.24 – 13.84, *P* = 0.02). A total of 61% of respondents were classified as having at least one manifestation of burnout. Having a religious affiliation was protective against one manifestation of burnout (OR = 0.43, 95% CI = 0.26 – 0.72, *P* = 0.001).

**Conclusions:**

Religion and spirituality are prominent and important aspects in the lives of many practicing internal medicine physicians in the USA and may impact physician well-being.

**Supplementary Information:**

The online version contains supplementary material available at 10.1007/s11606-025-09651-y.

## INTRODUCTION

Medicine is a profession steeped in meaning. The work of caring for patients is inherently meaningful, and the providers taking care of patients bring their own sources of meaning into the work that they do. Making meaning at work is important, as the loss of meaning at work has been associated with burnout,^1^ which has the potential to adversely affect patient safety,^2^ and a call to enhance meaning at work has been made by the National Academy of Medicine in their task force report^3^ on clinical burnout. Additionally, meaning and purpose are related to providers’ professional identities^4^ and have been shown^5^ to contribute to sustained engagement of providers with their vocational commitments, especially under adversity.

Spiritual and religious practices are one way in which providers make meaning in their lives and therefore in the work that they do. Spirituality has been defined in various ways by many scholars, but we, the authors, have come to understand it as the practice by which individuals search for and find meaning in something that lies outside of themselves. Spirituality therefore can encompass things such as music, nature, family, or a specific religion or deity. This understanding of spirituality has emerged from the authors’ personal experiences and interests in religion and spirituality and is shared here as a reflection of our personal reflexivity.^6^

The majority of physicians endorse a belief in God, and many physicians report that their spiritual and/or religious commitments were a reason they went into the field of medicine.^7^ There has been recent attention to the religious and spiritual commitments of physicians as they relate to topics such as self-care,^8^ physician conscience,^9^ and how these beliefs impact clinical practice.^10^

We thus assessed the religious and spiritual beliefs of practicing internists in the United States (US) using a national survey. We were also interested in testing the hypothesis that religious and spiritual practices are associated with reduced odds of burnout among internists.

## METHODS

### Study Design and Data Collection

We conducted a nationwide cross-sectional survey of internal medicine physicians practicing in the US.^11^ Between June 2023 and May 2024, paper surveys were sent to 1611 randomly selected internal medicine physicians identified through an American Medical Association membership database. Nationally, we oversampled hospitalists (30%) and Veterans Affairs internal medicine physicians (30%). The remaining 40% were internal medicine physicians in equal proportions (10% each) within the Northeast, Midwest, South, and West geographic regions of the US. Surveys that were returned as undelivered or those in which the respondent indicated they had retired were removed, resulting in a final sample of 1421 internal medicine physicians.

The well-validated methods developed by Dillman^12^ were used to distribute the survey: (1) a pre-notification email (when email address available) or letter; (2) an initial mailing information letter with a copy of the survey, a postage-paid return envelope, and a $20 incentive; (3) a reminder email or letter to non-respondents after approximately 2 weeks; and (4) additional survey mailings to non-respondents after 1 month, 2 months, and 3 months. We also included a link to an online version of the survey to accommodate those participants who preferred this method. Our full survey instrument is provided as a supplement.

Completed surveys did not include personally identifiable information and were anonymous. The study was reviewed and deemed exempt from regulation by the University of Michigan Institutional Review Board [HUM00228326].

### Study Measures

The survey included 42 questions (many with sub-questions) designed to assess a myriad of factors hypothesized to influence physician well-being and professional burnout. The survey assessed religious affiliation, levels of religious and spiritual practices and beliefs, and domains of well-being. We also collected physician demographic data including sex, race/ethnicity, marital and family status, and time in practice. We included questions on religiosity and spirituality from validated assessments as described in the Fetzer Institute Multidimensional Measurement of Religiousness/Spirituality for Use in Health Research.^13^ We assessed manifestations of burnout measured by the well-validated, 22-item Maslach Burnout Inventory-Human Services Survey (MBI-HSS)^14^ which assesses three domains of burnout: emotional exhaustion, depersonalization, and personal accomplishment. Consistent with prior research,^15^ a dichotomous outcome of at least one manifestation of burnout was defined as scoring high in the emotional exhaustion (i.e., ≥ 27) and/or depersonalization (i.e., ≥ 10) domains. Another dichotomous outcome of “extreme burnout” was defined as scores meeting set thresholds in all three burnout domains (i.e., emotional exhaustion ≥ 27, depersonalization ≥ 10, and personal accomplishment ≤ 33).

### Statistical Analysis

Descriptive statistics, proportions for categorical variables, and mean (SD) for continuous variables were calculated for physician characteristics. As a standard technique used to improve response quality and reduce response burden, respondents were not required to answer all questions. To investigate associations between internists’ religious and spiritual beliefs and practices and burnout, multivariable logistic regression was used. All regression models were adjusted for years of medical practice, sex, and race. Respondents were excluded from models if they had missing data for any of the included covariates in the respective models. A *P*-value < 0.05 was considered statistically significant. Stata SE 18.5 (College Station, TX) was used for all analyses.

## RESULTS

Of the 1421 physicians invited to participate, 629 (44.3%) completed a survey. Physician characteristics are provided in Table 1. A total of 60% of respondents were classified as having at least one manifestation of burnout (high emotional exhaustion and/or depersonalization), whereas 10% met thresholds for all three burnout domains (emotional exhaustion, depersonalization, and personal accomplishment). Of all respondents, 29.3% attend religious services at least once per month, 50.2% privately pray at least once per week other than in a place of worship, 69.7% believe in God or a higher power, 49.7% believe in a life after death, and 25.7% consider themselves to be very spiritual.

**Table 1:** Characteristics of Internal Medicine Physician Study Participants

Characteristics	*N* (%)
All	629
Median time spent in practice, years (IQR)	23 (15–29)
Median time spent working per week, hours (IQR)	50 (40–70)
Sex (*n* = 617)	
Male	376 (60.9)
Female	237 (38.4)
Prefer not to answer	4 (0.7)
Race/ethnicity (*n* = 611)	
White	362 (59.3)
Asian	189 (30.9)
Black or African American	31 (5.1)
Other	26 (4.3)
Native Hawaiian or Pacific Islander	2 (0.3)
American Indian or Alaska Native	1 (0.2)
Marital status (*n* = 618)	
Married or living as if married	534 (86.4)
Single, never married	33 (5.3)
Divorced	33 (5.3)
Separated	11 (1.8)
Widowed	7 (1.1)
Religious affiliation (*n* = 626)	
Protestant	123 (19.6)
Roman Catholic	122 (19.5)
None	107 (17.1)
Hindu	74 (11.8)
Other Christian	57 (9.1)
Jewish	38 (6.1)
Muslim	36 (5.8)
Other	36 (5.8)
Eastern Orthodox	17 (2.7)
Buddhist	16 (2.6)
Burnout (*n* = 622)	
One manifestation	379 (60.9)
Extreme	60 (9.6)

The distribution of religious affiliation was as follows: 19.6% Protestant, 19.5% Catholic, 17.1% None, 11.8% Hindu, 9.1% Other Christian, 6.1% Jewish, 5.8% Muslim, 5.8% Other, 2.7% Eastern Orthodox, 2.6% Buddhist. Spiritual and religious factors and levels of burnout by reported religious affiliation are presented in Table 2.

**Table 2:** Religiosity, Spirituality, and Burnout by Religious Affiliation

	**Protestant (*n* = 123)**	**Roman Catholic (*n* = 122)**	**None (*n* = 107)**	**Hindu (*n* = 74)**	**Other Christian (*n* = 57)**	**Jewish (*n* = 38)**	**Muslim (*n* = 36)**	**Other (*n* = 36)**	**Eastern Orthodox (*n* = 17)**	**Buddhist (*n* = 16)**
Attend religious services at least once a month	57 (46.3)	48 (39.3)	0 (0)	15 (20.3)	25 (43.9)	5 (13.2)	24 (66.7)	6 (16.7)	5 (29.4)	2 (12.5)
Privately pray at least once a week	75 (61.0)	79 (64.8)	9 (8.4)	44 (59.5)	35 (61.4)	8 (21.1)	30 (83.3)	21 (58.3)	8 (47.1)	6 (37.5)
Believe in God	97 (78.9)	100 (82.0)	26 (24.3)	62 (83.8)	50 (87.7)	19 (50.0)	34 (94.4)	26 (72.2)	14 (82.4)	9 (56.3)
Believe in life after death	76 (61.8)	83 (68.0)	11 (10.3)	34 (46.0)	40 (70.2)	7 (18.4)	33 (91.7)	15 (41.7)	7 (41.2)	11 (68.8)
Consider oneself to be very spiritual	44 (35.8)	32 (26.2)	8 (7.5)	23 (31.1)	18 (31.6)	6 (15.8)	14 (38.9)	12 (33.3)	4 (23.5)	3 (18.8)
One manifestation of burnout	77 (62.6)	74 (60.7)	82 (76.6)	32 (43.2)	42 (73.7)	19 (50.0)	17 (47.2)	17 (47.2)	10 (58.8)	9 (56.3)
Extreme burnout	10 (8.1)	11 (9.0)	13 (12.2)	4 (5.4)	8 (14.0)	2 (5.3)	1 (2.8)	5 (13.9)	1 (5.9)	5 (31.3)

Multivariable regression results highlighting demographic factors associated with religious and spiritual beliefs and practices are presented in Table 3. Respondents identifying as Black (or African-American) had four times greater odds of believing in God compared with non-Black respondents (OR = 4.14, 95% CI = 1.24–13.84, *P* = 0.02). A greater number of years practicing as an internist (as a proxy for older age) was associated with increased odds of privately praying at least once per week (OR = 1.02, 95% CI = 1.01–1.04, *P* = 0.01). We did not find statistically significant differences by sex in any of the religious or spiritual beliefs or practices assessed. Multivariable regression associations between religious and spiritual factors and internist burnout are presented in Table 4. After adjusting for sex, race, and years of practicing as an internist, we did not detect statistically significant associations between burnout and frequently attending religious services, frequently praying in private, belief in God, belief in life after death, or considering oneself a very spiritual person. We did, however, find that having a religious affiliation (vs. no affiliation) was protective against one manifestation of burnout (OR = 0.43, 95% CI = 0.26–0.72, *P* = 0.001).

**Table 3:** Multivariable Associations Between Demographic Factors and Religious/Spiritual Practices and Beliefs

	**OR**	**95% CI**	***P*-value**
**Attend religious services at least once per month**			
Black or African American (vs. all other reported races)	1.83	0.87–3.82	0.11
Male	1.22	0.85–1.76	0.28
Years practicing as an internist	1.01	0.99–1.03	0.23
**Privately pray at least once per week**			
Black or African American (vs. all other reported races)	1.92	0.90–4.09	0.09
Male	1.06	0.76–1.47	0.74
Years practicing as an internist	1.02	1.01–1.04	0.01
**Believe in God**			
Black or African American (vs. all other reported races)	4.14	1.24–13.84	0.02
Male	1.01	0.71–1.45	0.94
Years practicing as an internist	1.00	0.98–1.02	0.98
**Believe in life after death**			
Black or African American (vs. all other reported races)	1.62	0.77–3.40	0.20
Male	1.08	0.78–1.50	0.65
Years practicing as an internist	1.00	0.99–1.02	0.64
**Consider oneself to be very spiritual**			
Black or African American (vs. all other reported races)	1.37	0.63–2.99	0.42
Male	0.97	0.67–1.41	0.88
Years practicing as an internist	1.01	0.99–1.03	0.44

**Table 4:** Multivariable Associations Between Religiosity, Spirituality, and Internist Burnout

**Characteristic**	**One manifestation of burnout**			**Extreme burnout**		
	**OR**	**95% CI**	***P*-value**	**OR**	**95% CI**	***P*-value**
Attend religious services at least once per month	0.99	0.69–1.44	0.99	0.89	0.48–1.64	0.71
Privately pray at least once per week	0.99	0.71–1.40	0.97	0.83	0.48–1.46	0.52
Believe in God	0.89	0.61–1.29	0.53	1.13	0.61–2.09	0.70
Believe in life after death	0.94	0.67–1.31	0.71	1.61	0.91–2.83	0.10
Consider oneself to be very spiritual	0.92	0.63–1.35	0.67	0.80	0.42–1.54	0.50
Have a religious affiliation	0.43	0.26–0.72	0.001	0.74	0.37–1.49	0.40

All models adjusted for race, sex, and years of practice as an internist

## DISCUSSION

Our national survey results demonstrate that religion is a prominent aspect of US internists’ self-professed identities. The majority of our survey respondents endorsed a religious affiliation, and the majority of the physicians surveyed endorsed both a belief in God and a practice of praying at least once a week outside a place of worship. These results parallel those of the US population, although the religious diversity of our sample was broader than that of the US population. These results also align with what other researchers have found: US physicians’ religious identities are more diverse and are more likely to be affiliated with religions that are underrepresented in the US.

The implications of these findings as they relate to patient care and the vocation of medicine are manifold and intersect with domains of whole person care and physician well-being. Research has shown that many patients desire a discussion of religious and spiritual needs while hospitalized; however, many of those needs remain unmet.^16^ Prior work has suggested more fully addressing teaching spirituality and religion in medicine so that learners, regardless of any faith background, might have confidence in approaching such discussions.^17^ The physicians who are currently meeting those needs as they relate to whole person care are likely those who identify as religious. This is supported by the findings of one large national survey^18^ of US physicians that found that physicians who were more religious were more likely to report practices of addressing spirituality and religion in the clinical encounter. While our survey looked at the beliefs of US internists specifically, other studies have examined the self-reported religious identities across specialties^19^ and have found that the proportion of physicians in other specialties who hold religious and/or spiritual views varies widely. These findings have led some scholars to correctly point out that discussions with patients about religious and/or spiritual matters are therefore likely not equally distributed across the medical encounters.^19^

The way that a physician views the relationship between health and religion is also likely impacted by their own religious views. Research has revealed that physicians who identify as religious or spiritual are more likely to believe that religion and spirituality have a strong and positive impact on health.^20^ We found that the majority of our survey respondents prayed at least once a week outside of a place of worship. Prayer is a form of self-care and has been shown^21^ to be a coping resource to mitigate the adverse influences of stressful life situations on mental health. One can wonder if one of these places where physicians are praying is at work and how we can make space for people to do this comfortably.

Our study also found that having a religious affiliation, versus not, was protective against moderate burnout, after adjusting for sex, race, and years of practice as an internist. This finding is consistent with previous work that demonstrates that religion and spirituality may protect emergency medicine physicians^22^ against maladaptive behaviors caused by burnout and that internal medicine residents^23^ who were engaged in an active spiritual life were less susceptible to burnout. Other studies have shown that physicians are at increased risk of suicide^24^ compared to the general population. A potential piece of a solution to this crisis comes in the form of religious affiliation, as research has found that religious service attendance^25^ is associated with a lower risk of death from despair among health care professionals. The results from our survey provide additional insights on the potential protective mechanisms which should be promoted as a means of reducing burnout and its detrimental outcomes including death by suicide.

Black Americans have been found to have higher rates of religiosity than non-Black Americans^26^ and their beliefs often are more theistic^27^ (i.e., belief that God is more involved in the day-to-day workings of the world) than deistic. These theistic beliefs may influence how the physicians view adverse events like patient outcomes, difficult conflicts at work, and/or other challenges they face in their clinical work. In a lens of intersectionality, we know that Black physicians often face discrimination in the workplace^28^ and we know that historically people of faith have been discriminated against within medicine^29^ and that people with religious commitments often tend to feel that this identity has to be concealed^30^ within the sciences to avoid stigmatization. Given that Black respondents endorsed greater religious beliefs (specifically belief in God) in our survey, the inclusion and acceptance of non-White religious physicians may be doubly at risk. Efforts to include members of the scientific community who have faith claims have been called for.^31^

Our study has several limitations. First, the response rate, although in line with similar physician surveys, was 44.3%, which could bias our results if non-respondents were substantially different than respondents. Second, while we achieved representation from across the country, it is possible that the religious and spiritual beliefs and practices of our respondents may differ from those physicians who chose not to participate in the study. Third, although we examined numerous spiritual and religious factors thought to influence levels of burnout, our list of beliefs and practices was limited to what we collected in our surveys. Finally, given the cross-sectional design of our study, we could only show associations and not causation between religious and spiritual factors and internist characteristics, including burnout.

In a nationwide cross-sectional survey of internal medicine physicians practicing in the US, the majority of respondents claimed a religious identity and endorsed a belief in God and practices of prayer. Additionally, having a religious affiliation was protective against one manifestation of burnout. Given the protective mechanism of spirituality and religion in reducing burnout, efforts in the workplace directed at improving physician well-being should take into account the spiritual needs of physicians in addition to their physical, social, and emotional ones.

Our findings suggest that religion and spirituality play a large part in the lives of practicing US internists. The impacts that these beliefs and practices have on the lives of US physicians and their patients are important considerations as we consider our ongoing efforts as a profession around whole person care and physician well-being.

## Supplementary Information

Below is the link to the electronic supplementary material.

Supplementary file 1 (DOCX 195 KB)

## Data Availability

Deidentified data will be made available upon reasonable request and ethical approval (e-mail,mtgreene@med.umich.edu)

## Declarations

### Disclaimer

The funder had no role in the design, execution, analyses, interpretation of data, or decision to publish results.

## References

[1] **Abedini NC, Stack SW, Goodman JL, Steinberg KP.** “It's Not Just Time Off”: A Framework for Understanding Factors Promoting Recovery From Burnout Among Internal Medicine Residents. J Grad Med Educ 2018;10:26-32. 29467969 10.4300/JGME-D-17-00440.1 PMC5821021

[2] **Garcia C, Abreu L, Ramos J, et al.** Influence of Burnout on Patient Safety: Systematic Review and Meta-Analysis. Medicina 2019;55. 10.3390/medicina55090553 PMC6780563 31480365

[3] National Academy of Medicine. Taking Action Against Clinician Burnout: A Systems Approach to Professional Well-Being. Washington, DC 2019. 31940160

[4] **Tak HJ, Curlin FA, Yoon JD.** Association of Intrinsic Motivating Factors and Markers of Physician Well-Being: A National Physician Survey. J Gen Intern Med 2017;32:739-46. 28168540 10.1007/s11606-017-3997-y PMC5481224

[5] **Rushton CH.** Moral Resilience: Transforming Moral Suffering in Healthcare. New York, NY: Oxford University Press; 2018.

[6] **Olmos-Vega FM, Stalmeijer RE, Varpio L, Kahlke R.** A practical guide to reflexivity in qualitative research: AMEE Guide No. 149. Med Teach 2022;45:241-51. 10.1080/0142159X.2022.2057287 35389310

[7] **Robinson KA, Cheng M-R, Hansen PD, Gray RJ.** Religious and Spiritual Beliefs of Physicians. J Relig Health 2016;56:205-25. 10.1007/s10943-016-0233-8 27071796

[8] **Collier KM, James CA, Saint S, Howell J.** The Role of Spirituality and Religion in Physician and Trainee Wellness. J Gen Intern Med 2021;36:3199-201. 34109540 10.1007/s11606-021-06808-3 PMC8189548

[9] **Kennett J.** The Cost of Conscience. Camb Q Healthc Ethics 2016;26:69-81. 10.1017/S0963180116000657 27934571

[10] **Kørup AK, Søndergaard J, Lucchetti G, et al.** Religious values of physicians affect their clinical practice. Medicine 2019;98. 10.1097/MD.0000000000017265 PMC6756708 31568003

[11] **Houchens N, Greene MT, Sen S, et al.** Burnout Prevalence Among U.S. Internal Medicine Physicians: A Cross Sectional Study. Ann Intern Med. 2025 May 6. doi: 10.7326/ANNALS-24-02896. Epub ahead of print. 10.7326/ANNALS-24-02896 PMC12465106 40324201

[12] **Dillman DA.** Mail and Internet Surveys: The Tailored Design Method. 2nd ed. Hoboken, NJ: John Wiley & Sons Inc; 2007.

[13] The Fetzer Institute Multidimensional Measurement of Religiousness/Spirituality for Use in Health Research: A Report of the Fetzer Institute/National Institute on Aging Working Group. 2003. (Accessed 2025, April 8, at https://fetzer.org/resources/multidimensional-measurement-religiousnessspirituality-use-health-research.)

[14] **Maslach C, Jackson SE.** The measurement of experienced burnout. J Organ Behav 2007;2:99-113.

[15] **Rotenstein LS, Torre M, Ramos MA, et al.** Prevalence of Burnout Among Physicians. JAMA 2018;320. 10.1001/jama.2018.12777 PMC6233645 30326495

[16] **Williams JA, Meltzer D, Arora V, Chung G, Curlin FA.** Attention to Inpatients’ Religious and Spiritual Concerns: Predictors and Association with Patient Satisfaction. J Gen Intern Med 2011;26:1265-71. 21720904 10.1007/s11606-011-1781-y PMC3208457

[17] **Collier KM, James CA, Saint S, Howell JD.** Is It Time to More Fully Address Teaching Religion and Spirituality in Medicine? Ann Intern Med 2020;172:817-8. 32423346 10.7326/M20-0446

[18] **Curlin FA, Chin MH, Sellergren SA, Roach CJ, Lantos JD.** The Association of Physicians’ Religious Characteristics With Their Attitudes and Self-Reported Behaviors Regarding Religion and Spirituality in the Clinical Encounter. Med Care 2006;44:446-53. 16641663 10.1097/01.mlr.0000207434.12450.ef

[19] **Franzen AB.** Physicians in the USA: Attendance, Beliefs and Patient Interactions. J Relig Health 2015;54:1886-900. 25516296 10.1007/s10943-014-9986-0

[20] **Curlin FA, Sellergren SA, Lantos JD, Chin MH.** Physicians' Observations and Interpretations of the Influence of Religion and Spirituality on Health. Arch Intern Med 2007;167. 10.1001/archinte.167.7.649 PMC2867458 17420422

[21] **Lekhak N, Bhatta TR, Kahana E, Zauszniewski JA.** Prayer and Mental Health in Later Life: The Role of Positive Emotions. Issues Ment Health Nurs 2023;44:639-48. 37343582 10.1080/01612840.2023.2212776

[22] **Salmoirago-Blotcher E, Fitchett G, Leung K, et al.** An exploration of the role of religion/spirituality in the promotion of physicians' wellbeing in Emergency Medicine. Prev Med Rep 2016;3:189-95. 27419014 10.1016/j.pmedr.2016.01.009 PMC4929145

[23] **Doolittle BR, Windish DM, Seelig CB.** Burnout, Coping, and Spirituality Among Internal Medicine Resident Physicians. J Grad Med Educ 2013;5:257-61. 24404269 10.4300/JGME-D-12-00136.1 PMC3693690

[24] **Harvey SB, Epstein RM, Glozier N, et al.** Mental illness and suicide among physicians. Lancet 2021;398:920-30. 34481571 10.1016/S0140-6736(21)01596-8 PMC9618683

[25] **Chen Y, Koh HK, Kawachi I, Botticelli M, VanderWeele TJ.** Religious Service Attendance and Deaths Related to Drugs, Alcohol, and Suicide Among US Health Care Professionals. JAMA Psychiatry 2020;77. 10.1001/jamapsychiatry.2020.0175 PMC7203669 32374360

[26] **Mohamed B, Cox K, Diamant J, Gecewicz C.** Faith Among Black Americans: Pew Research Center; 2021 February 16.

[27] **Diamant J.** Three-quarters of Black Americans believe in God of the Bible or other holy scripture. Pew Research Center; 2021 March 24.

[28] **Laurencin CT, Murray M.** An American Crisis: the Lack of Black Men in Medicine. J Racial Ethn Health Disparities 2017;4:317-21. 28534304 10.1007/s40615-017-0380-y PMC5909952

[29] **Halperin EC.** The Jewish Problem in U.S. Medical Education, 1920-1955. J Hist Med Allied Sci 2001;56:140-67. 11392083 10.1093/jhmas/56.2.140

[30] **Barnes ME, Maas SA, Roberts JA, Brownell SE, Bolger MS.** Christianity as a Concealable Stigmatized Identity (CSI) among Biology Graduate Students. CBE Life Sci Educ 2021;20. 10.1187/cbe.20-09-0213 PMC8108500 33444108

[31] **Alexander DR, White RS.** Science profits most when people of faith feel equally welcomed. Nature 2024;630:305. 38862761 10.1038/d41586-024-01745-7

