# Supplementary material for: Spirituality and Religiosity of Internal Medicine Physicians in the USA: Results from a National Survey
Source: J Gen Intern Med. 2025 Jun 25;41(2):431–6. doi: 10.1007/s11606-025-09651-y (PMC12894608; doi:10.1007/s11606-025-09651-y)
Supplement: Supplementary file 1 — (DOCX 195 KB) [file 11606_2025_9651_MOESM1_ESM.docx]

**THE INTERNIST WELLNESS SURVEY**


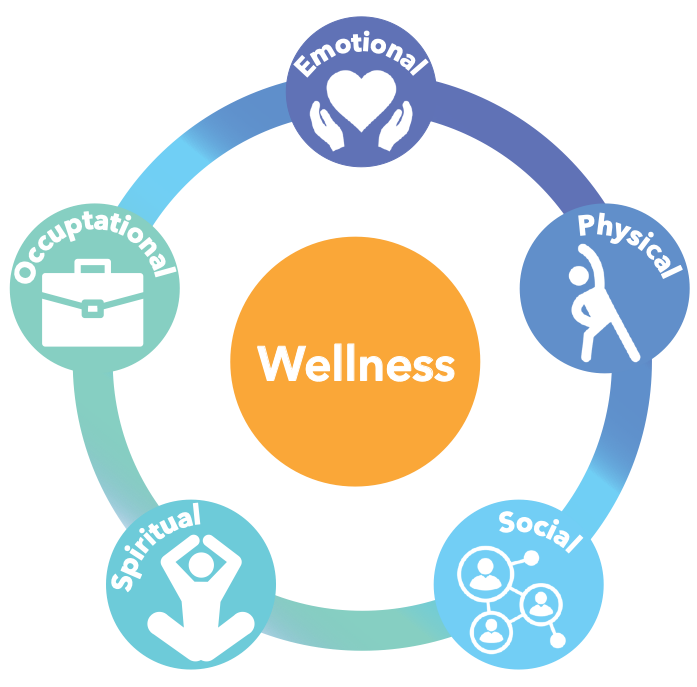


The purpose of this survey is to assess the level of wellness of internists across the United States. This study is being conducted by researchers from the University of Michigan.

Your participation in this survey is **completely voluntary** and should take **approximately 15 minutes**. By returning this survey, you are agreeing to participate in this research study. All responses will be kept anonymous and confidential. No identifying information will be collected. Your opinion is very valuable in helping us describe internist wellness patterns. Please use a blue or black pen to complete this survey.

**SECTION A - Mindfulness**

1. Below is a collection of statements about your everyday experience. Using the 1-6 scale below, please indicate how frequently or infrequently you currently have each experience. Please answer according to what really reflects your experience rather than what you think your experience should be. Please treat each item separately from every other item.

|  | **Almost Never** | **Very Infrequently** | **Somewhat Infrequently** | **Somewhat Frequently** | **Very Frequently** | **Almost Always** |
| --- | --- | --- | --- | --- | --- | --- |
| 1. It seems I am “running on automatic,” without much awareness of what I’m doing. | 1 | 2 | 3 | 4 | 5 | 6 |
| 1. I rush through activities without being really attentive to them. | 1 | 2 | 3 | 4 | 5 | 6 |
| 1. I find myself preoccupied with the future or the past. | 1 | 2 | 3 | 4 | 5 | 6 |
| 1. I find myself doing things without paying attention. | 1 | 2 | 3 | 4 | 5 | 6 |

1. How often do you practice any form of meditation (for example, breathing exercises, visualization, yoga)?

_1_ Every day

_2_ A few times per week

_3_ Once per week

_4_ A few times per month

_5_ Once per month

_6_ A few times per year

_7_ Never

1. How many years have you engaged in meditation?

_1_ Less than 1 year

_2_ 1-5 years

_3_ 6-10 years

_4_ >10 years

_5_ N/A – I do not practice meditation

**SECTION B – Religious/Spiritual Practices**

The next few questions are about your religious/spiritual practices.

1. How often do you attend religious services?

_1_ Never

_2_ Less than once a year

_3_ Several times a year

_4_ About once per month

_5_ 2 to 3 times per month

_6_ Nearly every week

_7_ Several times per week

1. What is your religious affiliation?

_1_ Buddhist

_2_ Hindu

_3_ Jewish

_4_ Muslim

_5_ Roman Catholic

_6_ Eastern Orthodox

_7_ Protestant

_8_ Other Christian

_9_ Other, please specify: ____________________________________

_10_ None

1. How often do you pray privately in places other than at church, synagogue or other place of worship?

_1_ Never

_2_ 1 to 2 times per month

_3_ 1 to 2 times per week

_4_ Approximately once per day

_5_ More than once per day

1. Do you believe in God or another higher power?

_1_ Yes

_2_  No

**Skip to question 8**

_3_ Undecided

_4_ Prefer not to answer

1. If yes, do you believe that God or another higher power is in control of the universe?

_1_ Yes

_2_  No

_3_ Undecided

_4_ Prefer not to answer

1. Do you believe there is a life after death?

_1_ Yes

_2_  No

_3_ Undecided

_4_ Prefer not to answer

1. To what extent do you consider yourself a spiritual person?

_1_ Not spiritual at all

_2_ Slightly spiritual

_3_ Moderately spiritual

_4_ Very spiritual

_5_ Prefer not to answer

1. Please indicate your level of agreement with the following statements:

|  | **Strongly agree** | **Somewhat agree** | **Neither agree nor disagree** | **Somewhat disagree** | **Strongly disagree** |
| --- | --- | --- | --- | --- | --- |
| 1. “I try hard to carry my religious beliefs over into all my other dealings in life.” | 1 | 2 | 3 | 4 | 5 |
| 1. “I have a strong sense of purpose in my **life**.” | 1 | 2 | 3 | 4 | 5 |
| 1. “I have a strong sense of purpose in my **work**.” | 1 | 2 | 3 | 4 | 5 |

1. “Sacred Moments” are described as deeply meaningful, memorable, and sometimes spiritual moments shared between physicians and patients – as if time stood still. These may happen spontaneously during times of crisis or sadness, or conversely during times of great joy.
   1. Have you ever experienced a Sacred Moment with a patient?

_1_ Yes

_2_  No **🡪 Skip to question 12**

- 1. If yes, how often do you experience Sacred Moments with a patient?

1 Weekly

2 Monthly

3 A few times per year

4 Yearly

5 A few times in my career

- 1. How often do you talk with your colleagues about your Sacred Moment experiences?

_1_ Never

_2_ Rarely

_3_ Sometimes

_4_ Often

_5_ Always

- 1. Please indicate how much you agree or disagree with the following statement. “Experiencing a Sacred Moment with a patient helps me feel less burned out.”

_1_ Strongly agree

_2_ Somewhat agree

_3_ Neither agree nor disagree

_4_ Somewhat disagree

_5_ Strongly disagree

**SECTION C – PHYSICAL AND SOCIAL ACTIVITIES**

The next section will ask questions about your participation in physical and social activities.

1. During the **last 7 days**, on how many days did you participate in any exercise or other physical activities?

________ **days**   No exercise or physical activities in the last 7 days **🡪 Skip to question 14**

1. How much time did you usually spend doing exercise or other physical activities on one of those days?

________ **hours** _________ **minutes per day**   Don’t know/Not sure

1. Within the **past 30 days**, indicate the number of times you’ve done any of the following.

|  | **Never** | **1 time** | **2 to 3 times** | **4 to 5 times** | **More than 5 times** |
| --- | --- | --- | --- | --- | --- |
| 1. Attended or participated in an in-person social event. | 1 | 2 | 3 | 4 | 5 |
| 1. Attended or participated in a virtual social event. | 1 | 2 | 3 | 4 | 5 |
| 1. Participated in an individual-based, relaxing event (e.g., read for pleasure, day spa, yoga, played a musical instrument). | 1 | 2 | 3 | 4 | 5 |
| 1. Participated in any other personal hobby. | 1 | 2 | 3 | 4 | 5 |
| 1. Did volunteer work. | 1 | 2 | 3 | 4 | 5 |

1. On a scale from 1-10, how much does work interfere with you participating in the social activities and hobbies you’d like to do?

1 2 3 4 5 6 7 8 9 10

Not at all All the time

**SECTION D –WELL-BEING**

1. Please indicate how much you agree or disagree with the following statements.

|  | **Strongly agree** | **Agree** | **Neither agree nor disagree** | **Disagree** | **Strongly disagree** |
| --- | --- | --- | --- | --- | --- |
| 1. My work schedule leaves me enough time for my personal/family life. | 1 | 2 | 3 | 4 | 5 |
| 1. I feel as if I can someday master my work. | 1 | 2 | 3 | 4 | 5 |
| 1. I have great autonomy over my work. | 1 | 2 | 3 | 4 | 5 |
| 1. I am paid less than I should be making. | 1 | 2 | 3 | 4 | 5 |

1. Below are 22 statements of job-related feelings. Please read each statement carefully and decide if you ever feel this way about your job. If you have *never* had this feeling, circle the number “0” (zero). If you have had this feeling, indicate *how often* you feel it by circling a number (from 1 to 6) that best describes how frequently you feel that way.

**Q17 Questions Redacted Due to a Survey Licensing Agreement.**

The 22 items came from the Maslach Burnout Inventory – Human Services Survey for Medical Personnel Copyright ©1981, 2016 Christina Maslach & Susan E. Jackson. All rights reserved in all media. Published by Mind Garden, Inc., www.mindgarden.com.

1. Overall, based on your definition of burnout, how would you rate your level of burnout?

_1_ I enjoy my work. I have no symptoms of burnout.

_2_ Occasionally I am under stress, and I don’t always have as much energy as I once did, but I don’t feel burned out.

_3_ I am definitely burning out and have one or more symptoms of burnout, such as physical and emotional exhaustion.

_4_ The symptoms of burnout that I’m experiencing won’t go away. I think about frustration at work a lot.

_5_ I feel completely burned out and often wonder if I can go on. I am at the point where I may need to seek some sort of help.

If you feel like you are experiencing feelings of burnout, there are resources available to help you. For example, the Physician Support Line is a free, confidential & anonymous service available Monday – Friday (except federal holidays) from 8:00 AM to 12:00 AM. **1 (888) 409-0141; https://www.physiciansupportline.com/**.

1. To what extent do you believe the following items contribute to **physician burnout**.

|  | **Not at all** | **A little bit** | **Somewhat** | **Quite a bit** | **A great deal** |
| --- | --- | --- | --- | --- | --- |
| 1. Concerns related to being sued for medical malpractice. | 1 | 2 | 3 | 4 | 5 |
| 1. Time devoted to engaging with health insurance companies. | 1 | 2 | 3 | 4 | 5 |
| 1. Issues working with the electronic health record. | 1 | 2 | 3 | 4 | 5 |
| 1. High workload (e.g., census is too high). | 1 | 2 | 3 | 4 | 5 |
| 1. Not having enough autonomy over workload. | 1 | 2 | 3 | 4 | 5 |
| 1. Not having the needed support (e.g., support staff and/or services). | 1 | 2 | 3 | 4 | 5 |
| 1. Feeling like you are not working at the top of your license. | 1 | 2 | 3 | 4 | 5 |
| 1. Feeling undervalued by senior leadership in my organization. | 1 | 2 | 3 | 4 | 5 |
| 1. Feeling undervalued by patients. | 1 | 2 | 3 | 4 | 5 |
| 1. Personal life stressors (e.g., childcare). | 1 | 2 | 3 | 4 | 5 |
| 1. Financial stressors. | 1 | 2 | 3 | 4 | 5 |
| 1. Feelings of being discriminated against by employer. | 1 | 2 | 3 | 4 | 5 |
| 1. Feelings of being discriminated against by patients. | 1 | 2 | 3 | 4 | 5 |

**SECTION E - SUPPORT**

1. To what extent do you agree or disagree with each of the following statements about your immediate PHYSICIAN supervisor? If you have more than one, please think about whomever supervises your clinical time.

|  | **Strongly disagree** | **Disagree** | **Neither agree nor disagree** | **Agree** | **Strongly agree** | **Do not know/**  **Not applicable** |
| --- | --- | --- | --- | --- | --- | --- |
| 1. My immediate physician supervisor empowers me to do my job. | 1 | 2 | 3 | 4 | 5 | 6 |
| 1. My immediate physician supervisor treats me with respect and dignity. | 1 | 2 | 3 | 4 | 5 | 6 |

1. How would you rank the level of support you receive from the following?

|  | **Poor** | **Fair** | **Good** | **Very good** | **Excellent** |
| --- | --- | --- | --- | --- | --- |
| 1. Your hospital’s senior leadership. | 1 | 2 | 3 | 4 | 5 |
| 1. Your coworkers. | 1 | 2 | 3 | 4 | 5 |
| 1. Your family. | 1 | 2 | 3 | 4 | 5 |
| 1. Your friends. | 1 | 2 | 3 | 4 | 5 |

1. Do you have any professional mentors?

_1_ Yes

_2_  No **🡪 Skip to question 23**

- 1. If yes, how would you rank the level of support you receive from your mentor(s)?

_1_ Poor

_2_ Fair

_3_ Good

_4_ Very good

_5_ Excellent

1. Do you believe that “human factors” – defined “as the scientific discipline concerned with the understanding of interactions among humans and other elements of a system in order to optimize human well-being and overall system performance” – has a role to play in enhancing your wellbeing as an internist?

_1_ Yes

_2_  No

_3_  Not sure

If yes, do you have any examples or suggestions for how? _______________________________________________________________________________________

______________________________________________________________________________________________________________________________________________________________________________

**SECTION F – WORK EXPERIENCE**

1. How long have you been practicing as an internist?

_____________ **Years**  _____________ **Months**

1. Please indicate below in which settings you perform clinical work. (Select only one)

_1_ Outpatient setting only

_2_  Inpatient setting only

_3_ Both the inpatient and outpatient setting

_4_  Other setting (please specify) _____________________________________

1. If you work in both the inpatient and outpatient setting, in which **setting** do you spend the majority of your clinical time? (Select only one)

_1_ Inpatient setting

_2_  Outpatient setting

_3_  Equal time in both settings

_4_  N/A - I do not work in both settings

1. In what **type of facility** do you spend the majority of your clinical time? (Select only one)

_1_ VA Medical Center or Clinic

_2_  Academic Medical Center or Clinic

_3_  Community Medical Center or Clinic

_4_ Other (please specify) __________________

1. How many hours do you work in a typical week (for hospitalists, please answer for a week when you are on service)? ___________
2. Of your total work hours, approximately what % of your time is spent on the following:

_______ Outpatient Care

_______ Inpatient Care

_______ Administrative Duties

_______ Research

_______ Teaching

_______ Other, please specify: _______________________

______Outpatient care

______Inpatient care

______Administrative duties

______Research

______Teaching

______Other

1. Do you consider yourself a hospitalist?

_1_ Yes

_2_  No

1. Do you provide primary care?

_1_ Yes

_2_  No **🡪 Skip to question 32**

- 1. If yes, what is your approximate patient panel size? _____________

| 1. In what state do you practice medicine? If more than one state, please indicate the one in which you practice the **most**. | ___________________  State |
| --- | --- |
| 1. In what country did you complete medical school? | ___________________  Country |

1. Has a friend or a close colleague of yours ever been sued for medical malpractice?

_1_ Yes

_2_  No

_3_  Don't Know

1. Have you ever been sued for medical malpractice?

_1_ Yes

_2_  No

1. Have malpractice concerns made you consider quitting medicine?

_1_ Yes

_2_  No

**SECTION G - DEMOGRAPHICS**

Lastly, we would like to know a little bit about you. Please answer the following questions.

1. Do you identify as transgender?

_1_ Yes

_2_  No

_3_ Prefer not to answer

1. What is your gender identity?

_1_ Male

_2_  Female

_3_ Non-binary

_4_ Other (please specify) ___________________________

_5_ Prefer not to answer

1. What is your race? (Check all that apply)

_1_ White

_2_ Black or African American

_3_ Asian

_4_ Native Hawaiian or Pacific Islander

_5_ American Indian or Alaskan Native

_6_ Other (please specify) __________________

1. Are you Hispanic or Latino?

_1_ Yes

_2_  No

1. What is your current marital status?

_1_ Single, never married

_2_ Married or living as if married

_3_  Separated

_4_ Widowed

_5_ Divorced

1. Do you have children under the age of 18 years?

_1_ Yes

_2_  No

**Thank you for completing The Internist Wellness Survey!**
